# Supplementary material for: Pyruvate Dehydrogenase Kinase 1 inhibition mediated oxidative phosphorylation enhancement in cartilage promotes osteoarthritis progression
Source: BMC Musculoskelet Disord. 2023 Jul 20;24:597. doi: 10.1186/s12891-023-06585-6 (PMC10357736; doi:10.1186/s12891-023-06585-6)
Supplement: Supplementary file 1 — Additional file 1. [file 12891_2023_6585_MOESM1_ESM.zip › MF.Gsea.1653627316242/GOMF_RNA_POLYMERASE_BINDING.html]

Details for gene set GOMF\_RNA\_POLYMERASE\_BINDING[GSEA]

|  || Dataset | 55457\_collapsed\_to\_symbols.cls.cls #OA\_versus\_normal.cls.cls #OA\_versus\_normal\_repos |
| Phenotype | cls.cls#OA\_versus\_normal\_repos |
| Upregulated in class | normal |
| GeneSet | GOMF\_RNA\_POLYMERASE\_BINDING |
| Enrichment Score (ES) | -0.42822704 |
| Normalized Enrichment Score (NES) | -1.5960008 |
| Nominal p-value | 0.015748031 |
| FDR q-value | 0.15522493 |
| FWER p-Value | 0.996 |
Table: GSEA Results Summary

  

Fig 1: Enrichment plot: GOMF\_RNA\_POLYMERASE\_BINDING      
 Profile of the Running ES Score & Positions of GeneSet Members on the Rank Ordered List

  

| SYMBOL | TITLE | RANK IN GENE LIST | RANK METRIC SCORE | RUNNING ES | CORE ENRICHMENT || 1 | ELP4 | elongator acetyltransferase complex subunit 4 [Source:HGNC Symbol;Acc:HGNC:1171] | 81 | 0.940 | 0.0466 | No |
| 2 | SMYD2 | SET and MYND domain containing 2 [Source:HGNC Symbol;Acc:HGNC:20982] | 856 | 0.553 | 0.0175 | No |
| 3 | SUPT4H1 | "SPT4 homolog, DSIF elongation factor subunit [Source:HGNC Symbol;Acc:HGNC:11467]" | 949 | 0.529 | 0.0401 | No |
| 4 | ERBB2 | erb-b2 receptor tyrosine kinase 2 [Source:HGNC Symbol;Acc:HGNC:3430] | 963 | 0.526 | 0.0687 | No |
| 5 | GTF2B | general transcription factor IIB [Source:HGNC Symbol;Acc:HGNC:4648] | 1315 | 0.455 | 0.0670 | No |
| 6 | URI1 | URI1 prefoldin like chaperone [Source:HGNC Symbol;Acc:HGNC:13236] | 1324 | 0.454 | 0.0919 | No |
| 7 | SCAF8 | SR-related CTD associated factor 8 [Source:HGNC Symbol;Acc:HGNC:20959] | 2207 | 0.337 | 0.0423 | No |
| 8 | SMYD3 | SET and MYND domain containing 3 [Source:HGNC Symbol;Acc:HGNC:15513] | 2361 | 0.319 | 0.0484 | No |
| 9 | PPIB | peptidylprolyl isomerase B [Source:HGNC Symbol;Acc:HGNC:9255] | 3372 | 0.214 | -0.0181 | No |
| 10 | AGO1 | argonaute RISC component 1 [Source:HGNC Symbol;Acc:HGNC:3262] | 3430 | 0.208 | -0.0109 | No |
| 11 | RPRD2 | regulation of nuclear pre-mRNA domain containing 2 [Source:HGNC Symbol;Acc:HGNC:29039] | 3472 | 0.205 | -0.0025 | No |
| 12 | RTRAF | "RNA transcription, translation and transport factor [Source:HGNC Symbol;Acc:HGNC:23169]" | 3752 | 0.181 | -0.0140 | No |
| 13 | ERCC5 | "ERCC excision repair 5, endonuclease [Source:HGNC Symbol;Acc:HGNC:3437]" | 4380 | 0.122 | -0.0559 | No |
| 14 | PCIF1 | phosphorylated CTD interacting factor 1 [Source:HGNC Symbol;Acc:HGNC:16200] | 5319 | 0.046 | -0.1262 | No |
| 15 | CDC73 | cell division cycle 73 [Source:HGNC Symbol;Acc:HGNC:16783] | 5604 | 0.023 | -0.1470 | No |
| 16 | PAF1 | "PAF1 homolog, Paf1/RNA polymerase II complex component [Source:HGNC Symbol;Acc:HGNC:25459]" | 5901 | 0.002 | -0.1699 | No |
| 17 | RPRD1A | regulation of nuclear pre-mRNA domain containing 1A [Source:HGNC Symbol;Acc:HGNC:25560] | 6294 | -0.027 | -0.1989 | No |
| 18 | CTR9 | "CTR9 homolog, Paf1/RNA polymerase II complex component [Source:HGNC Symbol;Acc:HGNC:16850]" | 6430 | -0.037 | -0.2073 | No |
| 19 | NEDD4 | NEDD4 E3 ubiquitin protein ligase [Source:HGNC Symbol;Acc:HGNC:7727] | 7407 | -0.111 | -0.2769 | No |
| 20 | RTF1 | "RTF1 homolog, Paf1/RNA polymerase II complex component [Source:HGNC Symbol;Acc:HGNC:28996]" | 7597 | -0.127 | -0.2845 | No |
| 21 | CCNT2 | cyclin T2 [Source:HGNC Symbol;Acc:HGNC:1600] | 8192 | -0.170 | -0.3211 | No |
| 22 | BRCA1 | BRCA1 DNA repair associated [Source:HGNC Symbol;Acc:HGNC:1100] | 8417 | -0.187 | -0.3280 | No |
| 23 | RPAP2 | RNA polymerase II associated protein 2 [Source:HGNC Symbol;Acc:HGNC:25791] | 8494 | -0.193 | -0.3231 | No |
| 24 | HNRNPU | heterogeneous nuclear ribonucleoprotein U [Source:HGNC Symbol;Acc:HGNC:5048] | 9847 | -0.306 | -0.4110 | Yes |
| 25 | YTHDC2 | YTH domain containing 2 [Source:HGNC Symbol;Acc:HGNC:24721] | 9872 | -0.309 | -0.3955 | Yes |
| 26 | BIN1 | bridging integrator 1 [Source:HGNC Symbol;Acc:HGNC:1052] | 10123 | -0.333 | -0.3962 | Yes |
| 27 | RECQL5 | RecQ like helicase 5 [Source:HGNC Symbol;Acc:HGNC:9950] | 10144 | -0.335 | -0.3789 | Yes |
| 28 | ANP32B | acidic nuclear phosphoprotein 32 family member B [Source:HGNC Symbol;Acc:HGNC:16677] | 10327 | -0.353 | -0.3731 | Yes |
| 29 | WAC | WW domain containing adaptor with coiled-coil [Source:HGNC Symbol;Acc:HGNC:17327] | 10514 | -0.375 | -0.3665 | Yes |
| 30 | PCF11 | PCF11 cleavage and polyadenylation factor subunit [Source:HGNC Symbol;Acc:HGNC:30097] | 10521 | -0.376 | -0.3458 | Yes |
| 31 | RRN3P1 | RRN3 pseudogene 1 [Source:HGNC Symbol;Acc:HGNC:30548] | 10582 | -0.382 | -0.3290 | Yes |
| 32 | DHX9 | DExH-box helicase 9 [Source:HGNC Symbol;Acc:HGNC:2750] | 10627 | -0.386 | -0.3107 | Yes |
| 33 | AGO2 | argonaute RISC catalytic component 2 [Source:HGNC Symbol;Acc:HGNC:3263] | 11246 | -0.463 | -0.3328 | Yes |
| 34 | RRN3 | "RRN3 homolog, RNA polymerase I transcription factor [Source:HGNC Symbol;Acc:HGNC:30346]" | 11762 | -0.543 | -0.3423 | Yes |
| 35 | TCERG1 | transcription elongation regulator 1 [Source:HGNC Symbol;Acc:HGNC:15630] | 11950 | -0.583 | -0.3240 | Yes |
| 36 | STOM | stomatin [Source:HGNC Symbol;Acc:HGNC:3383] | 12034 | -0.602 | -0.2966 | Yes |
| 37 | PKN2 | protein kinase N2 [Source:HGNC Symbol;Acc:HGNC:9406] | 12237 | -0.655 | -0.2754 | Yes |
| 38 | WDR43 | WD repeat domain 43 [Source:HGNC Symbol;Acc:HGNC:28945] | 12424 | -0.716 | -0.2496 | Yes |
| 39 | NCOA3 | nuclear receptor coactivator 3 [Source:HGNC Symbol;Acc:HGNC:7670] | 12455 | -0.727 | -0.2111 | Yes |
| 40 | PABPN1 | poly(A) binding protein nuclear 1 [Source:HGNC Symbol;Acc:HGNC:8565] | 12461 | -0.730 | -0.1704 | Yes |
| 41 | CCNT1 | cyclin T1 [Source:HGNC Symbol;Acc:HGNC:1599] | 12517 | -0.757 | -0.1321 | Yes |
| 42 | BRD4 | bromodomain containing 4 [Source:HGNC Symbol;Acc:HGNC:13575] | 12606 | -0.800 | -0.0939 | Yes |
| 43 | SCAF4 | SR-related CTD associated factor 4 [Source:HGNC Symbol;Acc:HGNC:19304] | 12827 | -1.021 | -0.0536 | Yes |
| 44 | ZFP36 | ZFP36 ring finger protein [Source:HGNC Symbol;Acc:HGNC:12862] | 12846 | -1.052 | 0.0042 | Yes |
Table: GSEA details [plain text format]

  

Fig 2: GOMF\_RNA\_POLYMERASE\_BINDING      
 Blue-Pink O' Gram in the Space of the Analyzed GeneSet

  

Fig 3: GOMF\_RNA\_POLYMERASE\_BINDING: Random ES distribution      
 Gene set null distribution of ES for **GOMF\_RNA\_POLYMERASE\_BINDING**
